# Convolutional neural network (CNN)-enabled electrocardiogram (ECG) analysis: a comparison between standard twelve-lead and single-lead setups

**DOI:** 10.3389/fcvm.2024.1327179

**Published:** 2024-02-15

**Authors:** Andrea Saglietto, Daniele Baccega, Roberto Esposito, Matteo Anselmino, Veronica Dusi, Attilio Fiandrotti, Gaetano Maria De Ferrari

**Affiliations:** ^1^ Division of Cardiology, Cardiovascular and Thoracic Department, “Citta della Salute e della Scienza” Hospital, Turin, Italy; ^2^ Department of Medical Sciences, University of Turin, Turin, Italy; ^3^ Department of Computer Science, University of Turin, Turin, Italy; ^4^ Laboratorio InfoLife, Consorzio Interuniversitario Nazionale per l'Informatica (CINI), Rome, Italy

**Keywords:** artificial intelligence, deep learning, electrocardiogram, single-lead, screening

## Abstract

**Background:**

Artificial intelligence (AI) has shown promise in the early detection of various cardiac conditions from a standard 12-lead electrocardiogram (ECG). However, the ability of AI to identify abnormalities from single-lead recordings across a range of pathological conditions remains to be systematically investigated. This study aims to assess the performance of a convolutional neural network (CNN) using a single-lead (D1) rather than a standard 12-lead setup for accurate identification of ECG abnormalities.

**Methods:**

We designed and trained a lightweight CNN to identify 20 different cardiac abnormalities on ECGs, using data from the PTB-XL dataset. With a relatively simple architecture, the network was designed to accommodate different combinations of leads as input (<100,000 learnable parameters). We compared various lead setups such as the standard 12-lead, D1 alone, and D1 paired with an additional lead.

**Results:**

The CNN based on single-lead ECG (D1) achieved satisfactory performance compared to the standard 12-lead framework (average percentage AUC difference: −8.7%). Notably, for certain diagnostic classes, there was no difference in the diagnostic AUC between the single-lead and the standard 12-lead setups. When a second lead was detected in the CNN in addition to D1, the AUC gap was further reduced to an average percentage difference of −2.8% compared with that of the standard 12-lead setup.

**Conclusions:**

A relatively lightweight CNN can predict different classes of cardiac abnormalities from D1 alone and the standard 12-lead ECG. Considering the growing availability of wearable devices capable of recording a D1-like single-lead ECG, we discuss how our findings contribute to the foundation of a large-scale screening of cardiac abnormalities.

## Introduction

The 12-lead electrocardiogram (ECG) is a fundamental instrument used in diagnosing cardiac abnormalities. Traditionally, 12-lead ECGs are analyzed by trained medical professionals; however, recent advances in artificial intelligence (AI) and, in particular, deep neural networks (1) have enabled methods to accurately analyze ECGs (2, 3). In the diagnosis of rhythm disturbances, these approaches outperform expert cardiologists in terms of diagnostic accuracy (4). Furthermore, AI demonstrated the ability to recognize specific patterns and ECG waveform abnormalities that are invisible to the human eye, e.g., detecting a high likelihood of cardiac contractile dysfunction or a future or past episode of atrial fibrillation from an “apparently” normal ECG in sinus rhythm (5, 6). Thus, an AI-based analysis of the 12-lead ECG has the potential for a prompt and accurate diagnosis of ECG abnormalities and early detection of different cardiac diseases. The rapid adoption of wearable devices capable of recording single-lead ECGs (7–10) has opened new opportunities in diagnosing cardiac disorders such as atrial fibrillation. Anecdotally, a precordial smartwatch has been found to detect signs of myocardial ischemia with a 12-lead ECG and to record an event of ventricular fibrillation (11). AI-based algorithms have shown potential in detecting cardiac alterations from single-lead ECG recordings. For example, Attia et al. (12) reported that an AI-based analysis of a single-lead ECG of a smartwatch worn by approximately 2,500 patients correctly identified a left ventricular ejection fraction (LVEF) of <40% in 16 patients. To the best of our knowledge, there are no studies that systematically assess the performance of AI-based algorithms based on single-lead ECG across different ECG cardiac diagnoses.

Moreover, the computing ability of battery-operated wearable devices is typically limited, making large deep neural networks not suitable for such platforms and necessitating the development of *ad hoc* algorithms for ECG analysis. This work aims to address the above issues through a twofold contribution. First, we propose an *ad hoc* method for detecting cardiac abnormalities based on a lightweight CNN, as opposed to the state-of-the-art architectures typically used for this task. Second, we train our CNN to identify from the single-lead D1 (and in combination with D2) more than 20 different cardiac conditions that are typically diagnosed from standard 12-lead ECGs with far more complex architectures. We show that for multiple ECG abnormalities [e.g., AV block, complete left or right bundle branch block, and lateral myocardial infarction (LMI)], the performance of our single-lead lightweight CNN is comparable to that provided by more complex architectures using standard 12-lead setups. These results show the potential to integrate AI-based algorithms into wearable devices for mass-screening the population against cardiac diseases, as discussed in the last section of this article.

## Methods

### The PTB-XL ECG dataset

The PTB-XL ECG dataset (13, 14) is a publicly available dataset containing 21,837 clinical 12-lead ECGs (based on the Wilson lead system) of 10 s length from 18,885 patients (52% males, 48% females, median age of 62 years; interquartile range, 22 years; range, 0–95 years) recorded at a sampling frequency of 500 Hz.

**Figure 1:**
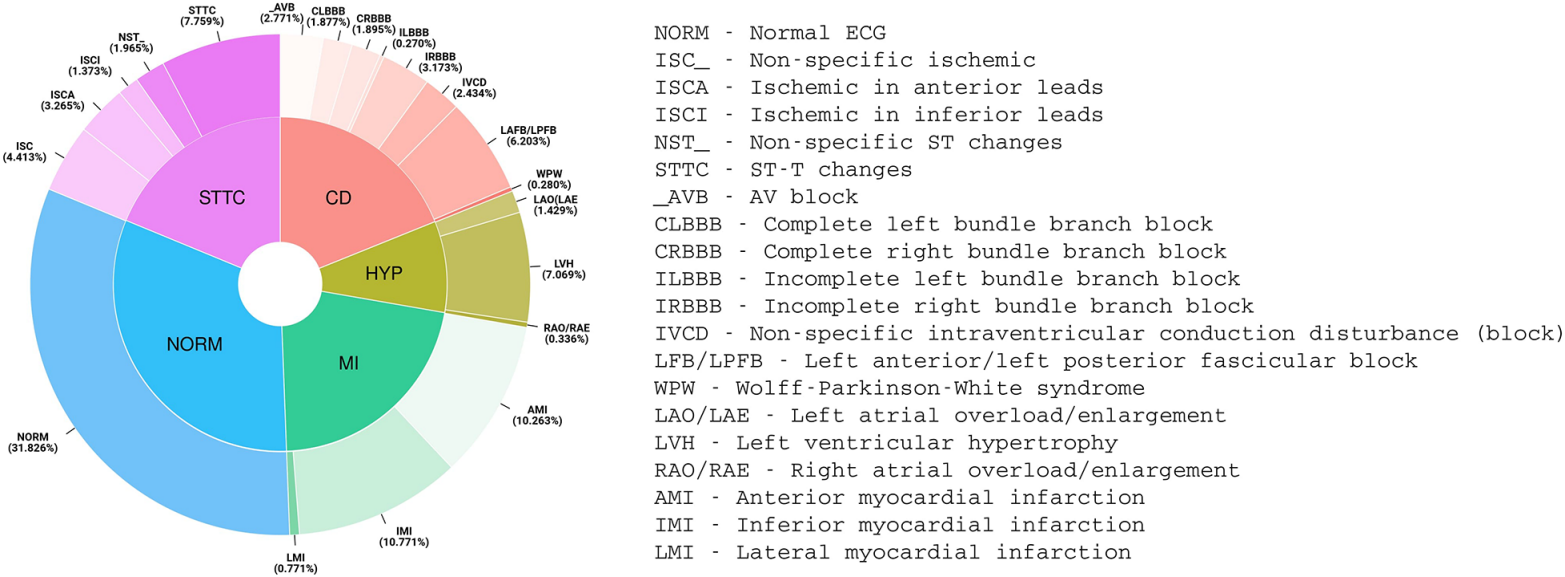
Distribution of PTB-XL ECG diagnoses (aggregated into 20 diagnostic classes and 5 diagnostic superclasses).

Prediction was assessed as a multi-label classification task (13) according to the 20 diagnostic classes shown in Figure 1 (the 5 macro classes are reported only for the sake of completeness and were not considered in this study).

The ECGs were divided into 10 folds. According to Wagner et al. (13), since containing only ECGs validated by at least one cardiologist and therefore presumably representative of the highest label quality, the ninth fold was used as the validation set and the tenth fold as the test set. The other eight folds served as the training set.

### Data preprocessing

As in previous literature, ECGs were downsampled at 100 Hz signals (1,000 samples for each of the 12 leads, for each ECG). ECGs that either had (i) conflicting labels (being classified as NORM and as some other non-NORM diagnostic class), (ii) lack of classification (not classified into any diagnostic class), or (iii) diagnostic statements with a likelihood equal to 0% were filtered out. Of the resulting 21,008 ECGs, 17,598 were used as the training set, 1,708 as the validation set, and 1,702 as the test set. Following standard practice, we independently normalized each lead over the mean and standard deviation computed on the training set.

Finally, each ECG was represented as a matrix with *L* rows and *W* columns, where *L* represents the number of considered leads (between 1 and 12, depending on the number of leads input) and *W* represents the length of the ECG (maximum of 1,000 samples at 100 Hz sampling frequency). The number of samples that was provided as input to the CNN eventually depended on the network receptive field, as discussed below.

### Convolutional network architecture

**Figure 2:**
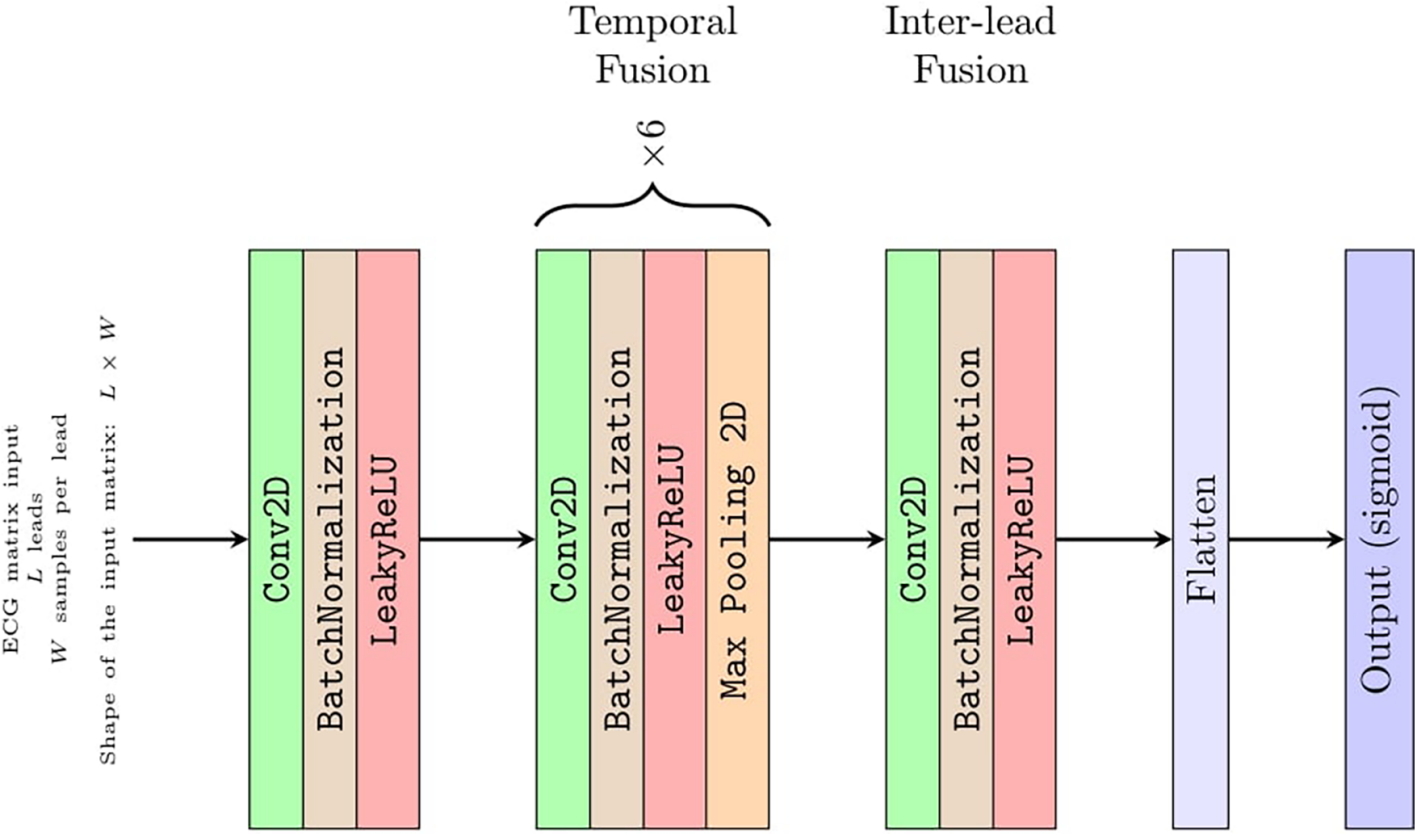
Detailed CNN architecture.

Although Strodthoff et al. (15) found the deep ResNet with 101 layers was the best-performing model, to assess CNN diagnostic performance for an arbitrary number of *L* input leads, we used a straightforward architecture with eight convolutional layers (Figure 2). This architecture is composed of a few sections with well-defined functions that can be easily interpreted (6). Keras framework with a TensorFlow (Google; Mountain View, CA, USA) backend was used to implement the CNN. In the first section, a convolutional layer with one 1 × 1 filter performs a preliminary linear transformation over the *L* leads taken as input. Next, a section with six convolve–normalize–pool blocks follows. For each block, a convolutional layer extracts feature maps, where batch normalization is meant to accelerate learning and a 1 × 2 MaxPooling operator halves the feature maps size across the temporal axis *W* only. The number of output feature maps by each convolutional block is 16, 16, 32, 32, 64, and 64, respectively. The filter sizes are 1 × 5, 1 × 5, 1 × 5, 1 × 3, 1 × 3, and 1 × 3, respectively. Attia et al. (6) reported that convolutional filters have a dilation rate of 2 to increase the receptive field without resorting to 4× pooling. This section of the CNN fuses each lead over the temporal axis and projects the *L* leads over a larger feature space. The output of this section is a vector of 64 feature maps with a size of 1 × 1, i.e., a vector of 64 features, for each of the *L* leads. Since the receptive field of these features was *W* = 344 samples along the temporal axis, this network required about 3.4 s of ECG sampling at 100 Hz to produce a diagnostic output. Thus, the presence of 10 s of sampling of each ECG allowed for a data augmentation strategy as reported below. Notably, each lead was independently filtered up to this point, i.e., there was no mixing between information coming from different leads.

In the third section, an eighth and last convolutional layer with 128 filters with a size of 1 × 1 projects a linear combination of these features over a larger 128 × 1 vector of features. This was the only part of the network where information from different leads was fused.

The output layer is composed of 20 neurons with sigmoid activations, one for each of the 20 PTB-XL ECG diagnostic classes, acting as 20 independent binary classifiers.

The number of learnable parameters of this CNN depends on the number of leads provided in input (i.e., from dimension *L* of the input matrix introduced below) and ranges from 36,000 parameters for *L* = 1 up to 96,000 parameters for *L* = 12 leads.

### Data augmentation

To maximize the ability of the network to generalize over data unseen at training time, the training samples were augmented with the following random transformations:

• Gaussian noise: noise, drawn from a Gaussian distribution with zero mean and standard deviation from a uniform distribution in the [0.01, 0.1] interval, was added to each ECG lead.
• Time scale: for each ECG, all leads were temporally stretched or compressed by a random factor uniformly drawn from the [0.8, 1.2] interval.
• Amplitude: for each ECG, all the samples were multiplied by a random factor uniformly drawn from the [0.7, 1.3] interval.
• Temporal cropping: starting from a random position, in each ECG, a window of *W* = 344 consecutive samples was cropped. The width of the window corresponds to the receptive field of the above CNN.

Notably, these transformations were applied only to the training set except for temporal cropping that was applied to all: the network was trained and evaluated on random segments of about 3.4 s.

### Input setups

The CNN was designed to receive as input a matrix of size *L* × *W*, where *L* represents an arbitrary number of leads between 1 and 12. Regardless of *L*, the network always produced a vector y of 20 values ∈ [(0, 1)] as output, corresponding to the diagnostic classes. Hence, the network allowed us to fairly compare experiments where different subsets of leads act as input of the network. In total, we assessed four different input setups:

• Standard 12-lead ECG, input matrix 12 × 344 (i.e. 12 leads of 344 samples at 100 Hz each, which is about 3.4s of ECG)r
• Independent leads (eight-lead) ECG, input matrix 8 × 344; considering that four leads (D3, aVR, aVL, aVF) are linear combinations of leads D1 and D2, the eight independent leads (lead D1, lead D2, leads V1–V6) were used as input.
• Single-lead ECG (lead D1), input matrix 1 × 344.
• Two-lead ECG (lead D1 + additional lead), input matrix 2 × 344; lead D1 was coupled with a second lead, registering information from a different spatial axis.

### Training procedure and performance assessment

The network was trained to minimize the sum of the binary cross-entropies computed over the 20 outputs of the network. Adam (16) was used as an optimizer. The learning rate was set to decay linearly from 10^−2^ to 10^−4^ by a batch size equal to 32 ECGs for 200 epochs of training in total.

According to Wagner et al. (13), the results were reported as macro-averaged and threshold-free measures; in particular, the performance of the network was evaluated by the area under the curve (AUC) (13). In addition, we also evaluated the sensitivity and specificity at the best threshold identified by the receiver–operator curve (ROC) analysis for each diagnostic class (please refer to Supplementary Tables S4 and S5).

To evaluate the consistency of our results, we also tested a fine-tuned version of the trained network on two other publicly available datasets, namely, the Georgia and China datasets (14). Please refer to the Supplementary Material for a more detailed description of this additional evaluation.

## Results

Table 1 summarizes the AUC with a 95% CI (over 50 runs) of the PBT-XL ECG test set for each diagnostic class and the inter-class average, for each investigated diagnostic scenario. As a state-of-the-art reference, the table also presents the outputs using the architecture (deep ResNet with 101 layers) proposed by Strodthoff et al (15).

**Table 1:** Average AUC with 95% CI (over 50 runs) of the considered scenarios.

Classes	D1	D1 + D2	D1 + V1	D1 + V2	D1 + V3	D1 + V4	D1 + V5	D1 + V6	8 leads	12 leads	12 w/o aug	Ref
NORM	89.76 [89.69, 89.83]	93.95 [93.91, 93.99]	92.00 [91.94, 92.06]	91.91 [91.84, 91.98]	92.23 [92.17, 92.29]	92.90 [92.86, 92.94]	93.07 [93.01, 93.13]	92.79 [92.75, 92.83]	96.06 [96.02, 96.10]	**96.07** [96.03, 96.11]	96.06 [95.96, 96.16]	95.2
ISC_	90.73 [90.64, 90.82]	92.96 [92.88, 93.04]	91.81 [91.72, 91.90]	91.68 [91.58, 91.78]	91.64 [91.54, 91.74]	93.46 [93.38, 93.54]	95.18 [95.10, 95.26]	95.57 [95.50, 95.64]	95.28 [95.19, 95.37]	95.40 [95.31, 95.49]	95.10 [94.96, 95.24]	**96**.**5**
ISCA	87.94 [87.81, 88.07]	87.77 [87.65, 87.89]	89.11 [88.98, 89.24]	88.76 [88.62, 88.90]	89.68 [89.53, 89.83]	89.74 [89.60, 89.88]	89.44 [89.29, 89.59]	88.47 [88.34, 88.60]	91.85 [91.70, 92.00]	91.97 [91.80, 92.14]	90.38 [90.00, 90.76]	**93**.**4**
ISCI	72.01 [71.37, 72.65]	91.68 [91.40, 91.96]	71.75 [71.17, 72.33]	77.32 [76.75, 77.89]	76.59 [76.04, 77.14]	79.16 [78.64, 79.68]	81.60 [81.16, 82.04]	81.90 [81.42, 82.38]	92.68 [92.40, 92.96]	**93.85** [93.48, 94.22]	90.72 [90.16, 91.28]	91.5
NST_	84.86 [84.59, 85.13]	87.11 [86.86, 87.36]	85.09 [84.82, 85.36]	84.60 [84.29, 84.91]	84.86 [84.64, 85.08]	84.97 [84.71, 85.23]	87.32 [87.07, 87.57]	86.57 [86.30, 86.84]	**88.24** [88.03, 88.45]	88.23 [87.96, 88.50]	86.92 [86.45, 87.39]	86.7
STTC	80.73 [80.58, 80.88]	84.81 [84.69, 84.93]	81.97 [81.84, 82.10]	84.52 [84.38, 84.66]	85.87 [85.76, 85.98]	87.53 [87.42, 87.64]	88.29 [88.19, 88.39]	86.53 [86.43, 86.63]	90.11 [90.00, 90.22]	90.58 [90.48, 90.68]	89.60 [89.45, 89.75]	**91**.**1**
_AVB	92.50 [92.21, 92.79]	95.20 [95.09, 95.31]	93.27 [93.09, 93.45]	92.97 [92.79, 93.15]	93.21 [93.06, 93.36]	93.18 [92.97, 93.39]	93.59 [93.48, 93.70]	93.60 [93.38, 93.82]	94.24 [94.09, 94.39]	93.73 [93.58, 93.88]	93.75 [93.55, 93.95]	**96**.**9**
CLBBB	99.56 [99.52, 99.60]	99.67 [99.65, 99.69]	99.75 [99.72, 99.78]	99.76 [99.74, 99.78]	99.68 [99.66, 99.70]	99.65 [99.62, 99.68]	99.72 [99.71, 99.73]	99.71 [99.69, 99.73]	99.56 [99.51, 99.61]	99.52 [99.46, 99.58]	99.20 [99.08, 99.32]	**99**.**9**
CRBBB	98.94 [98.84, 99.04]	99.34 [99.31, 99.37]	99.69 [99.68, 99.70]	99.58 [99.56, 99.60]	99.22 [99.14, 99.30]	99.16 [99.10, 99.22]	99.24 [99.20, 99.28]	99.16 [99.11, 99.21]	99.58 [99.56, 99.60]	99.56 [99.54, 99.58]	99.60 [99.58, 99.62]	**99**.**8**
ILBBB	90.48 [89.92, 91.04]	90.74 [90.26, 91.22]	92.07 [91.70, 92.44]	93.07 [92.71, 93.43]	91.18 [90.55, 91.81]	93.39 [92.89, 93.89]	**93.72** [93.22, 94.22]	92.87 [92.13, 93.61]	92.32 [91.73, 92.91]	91.47 [90.87, 92.07]	87.43 [86.34, 88.52]	91.9
IRBBB	73.86 [73.48, 74.24]	79.14 [78.82, 79.46]	96.17 [96.09, 96.25]	91.41 [91.25, 91.57]	76.17 [75.91, 76.43]	75.33 [75.03, 75.63]	76.07 [75.77, 76.37]	79.75 [79.46, 80.04]	95.90 [95.78, 96.02]	96.16 [96.03, 96.29]	95.02 [94.79, 95.25]	**98**.**0**
IVCD	68.41 [68.10, 68.72]	73.86 [73.57, 74.15]	71.46 [71.17, 71.75]	72.52 [72.24, 72.80]	71.41 [71.14, 71.68]	69.89 [69.62, 70.16]	68.94 [68.60, 69.28]	70.82 [70.51, 71.13]	75.47 [75.23, 75.71]	76.40 [76.03, 76.77]	**78.52** [78.14, 78.90]	74.4
LAFB/LPFB	79.01 [78.78, 79.24]	97.57 [97.51, 97.63]	83.56 [83.40, 83.72]	80.94 [80.72, 81.16]	81.72 [81.55, 81.89]	85.87 [85.71, 86.03]	89.58 [89.48, 89.68]	90.86 [90.74, 90.98]	97.83 [97.76, 97.90]	**98.20** [98.15, 98.25]	97.67 [97.59, 97.75]	97.5
WPW	86.55 [85.44, 87.66]	84.40 [83.16, 85.64]	90.70 [89.56, 91.84]	84.33 [82.85, 85.81]	85.12 [83.96, 86.28]	84.68 [83.69, 85.67]	83.07 [81.55, 84.59]	83.02 [81.31, 84.73]	92.38 [91.40, 93.36]	**93.36** [92.22, 94.50]	90.26 [88.81, 91.71]	85.5
LAO/LAE	80.60 [80.19, 81.01]	**86.93** [86.57, 87.29]	83.52 [83.08, 83.96]	80.27 [79.74, 80.80]	79.92 [79.55, 80.29]	81.30 [80.83, 81.77]	82.16 [81.70, 82.62]	81.90 [81.44, 82.36]	81.22 [80.59, 81.85]	82.43 [81.75, 83.11]	80.37 [79.64, 81.10]	78.2
LVH	87.97 [87.85, 88.09]	90.77 [90.62, 90.92]	91.42 [91.27, 91.57]	90.92 [90.76, 91.08]	90.98 [90.84, 91.12]	92.06 [91.92, 92.20]	93.23 [93.10, 93.36]	94.08 [93.98, 94.18]	94.44 [94.33, 94.55]	94.24 [94.11, 94.37]	95.10 [94.95, 95.25]	**95**.**3**
RAO/RAE	84.93 [84.14, 85.72]	**97.11** [96.83, 97.39]	87.60 [86.83, 88.37]	84.21 [83.34, 85.08]	80.86 [79.63, 82.09]	88.83 [87.83, 89.83]	89.35 [88.56, 90.14]	89.41 [88.62, 90.20]	93.81 [93.15, 94.47]	92.55 [91.83, 93.27]	92.02 [91.01, 93.03]	95.9
AMI	83.18 [83.03, 83.33]	85.67 [85.57, 85.77]	90.29 [90.18, 90.40]	94.94 [94.87, 95.01]	94.16 [94.09, 94.23]	90.17 [90.04, 90.30]	86.26 [86.16, 86.36]	86.35 [86.21, 86.49]	96.70 [96.64, 96.76]	96.72 [96.64, 96.80]	96.21 [96.06, 96.36]	**96**.**9**
IMI	71.59 [71.23, 71.95]	93.28 [93.16, 93.40]	75.18 [74.96, 75.40]	74.34 [74.06, 74.62]	75.40 [75.18, 75.62]	77.05 [76.78, 77.32]	80.26 [80.04, 80.48]	83.05 [82.84, 83.26]	93.05 [92.93, 93.17]	**95.16** [95.08, 95.24]	95.11 [94.97, 95.25]	94.6
LMI	98.81 [98.58, 99.04]	**99.59** [99.51, 99.67]	98.61 [98.40, 98.82]	99.17 [99.01, 99.33]	99.29 [99.10, 99.48]	99.36 [99.26, 99.46]	99.36 [99.27, 99.45]	99.58 [99.51, 99.65]	98.25 [97.62, 98.88]	99.08 [98.86, 99.30]	95.88 [94.72, 97.04]	91.4
Avg	85.12 [85.00, 85.24]	90.58 [90.50, 90.66]	88.25 [88.16, 88.34]	87.86 [87.76, 87.96]	86.96 [86.86, 87.06]	87.88 [87.80, 87.96]	88.47 [88.36, 88.58]	88.80 [88.68, 88.92]	92.95 [92.85, 93.05]	**93.23** [93.15, 93.31]	92.25 [92.08, 92.42]	93.10

The last column represents the results of the best architecture proposed by Strodthoff et al. (15). The AUCs are reported as percentages. The bold numbers correspond to the best performer for each class. The underlined numbers correspond to the competitive results.

### Standard (12-lead) setup

The average AUC across the 20 diagnostic classes achieved by the standard 12-lead setup was 93.2%, and when the data augmentation strategy was omitted during CNN training, the performance dropped by 1.1%.

### Independent (eight-lead) setup

The eight-lead setup (leads D1, D2, V1, V2, V3, V4, V5, and V6) did not induce a performance loss as compared with the 12-lead setup: the average percentage difference over the 20 diagnostic classes was −0.3%. Being the four excluded leads (D3, aVR, aVL, aVF), which are linear combinations of leads D1 and D2, this finding supports the idea that they provide redundant information, which is associated with a computational burden (96,000 and 56,000 parameters in the 12- and 8-lead setups, respectively).

### Single-lead (D1) setup

**Figure 3:**
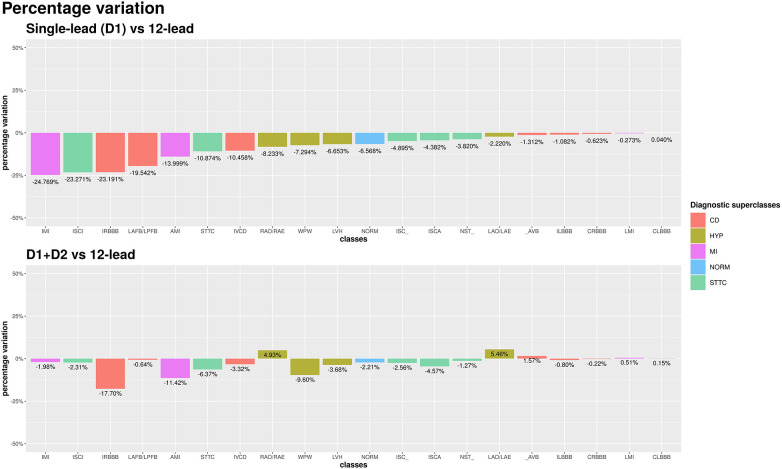
AUC percentage variation among single-lead (D1) and 12-lead scenarios (top chart) and among D1 + D2 and 12-lead scenarios (bottom chart). Percentage differences are reported relative to the 12-lead setup.

The single D1-lead setup was associated with an average of −8.7% accuracy as compared with the 12-lead setup. For some specific diagnostic classes, the performance drop was significant. Namely, inferior myocardial infarction (IMI), inferior myocardial ischemia (ISCI), and incomplete right bundle branch block (IRBBB) showed a drop in accuracy of >20%. On the other hand, for several other diagnostic classes, non-specific ischemic changes (ISC_), ischemic changes in anterior leads (ISCA), non-specific ST changes (NST_), AV block (_AVB), complete left bundle branch block (CLBBB), complete right bundle branch block (CRBBB), incomplete left bundle branch block (ILBBB), left atrial overload/enlargement (LAO/LAE), and LMI, the drop in accuracy was <5% (the performance drop was smaller than 1% for CLBBB, CRBBB, and LMI). The upper panel of Figure 3 shows the performance comparison of single-lead and standard 12-lead setups.

### Two-lead setup

Among all leads, D2 yielded the best increase in average AUC compared to the single D1-lead setup. The average AUC across the 20 diagnostic classes achieved by the two-lead setup was 90.6%, which represented an average percentage difference over the 20 diagnostic classes of −2.8% compared with the standard 12-lead setup (90.6% vs. 93.2%). The use of an additional lead proved particularly useful in the diagnostic classes in which the single-lead setup performed worse. In the IMI and SCI classes, the accuracy loss compared to standard 12-lead ECG was reduced from >20% with single-lead to <2% with a two-lead setup. The bottom panel of Figure 3 shows the performance comparison of two-lead vs. standard 12-lead setups.

Interestingly, for two diagnostic classes, namely, right atrial overload/enlargement (RAO/RAE) and LAO/LAE, the two-lead performed better than the 12-lead setup (+4.9% and +5.5%, respectively). On the other hand, the diagnosis of anterior (not anterolateral) myocardial infarction still showed a 10% lower accuracy with the two-lead as compared with the 12-lead setup (in this case, the addition of V2, a lead recording information from a different spatial axis, reduced the diagnostic loss to <2%).

### Validation on an external dataset

We fine-tuned the trained network to test it on other two external freely available datasets, namely, the Georgia and China datasets (14), to increase the rigor. The drops in the average AUCs calculated on the test set of these datasets are in agreement with the findings on the PTB-XL dataset. Please refer to the Supplementary Material for a more detailed description of this additional evaluation.

## Discussion

**Figure 4:**
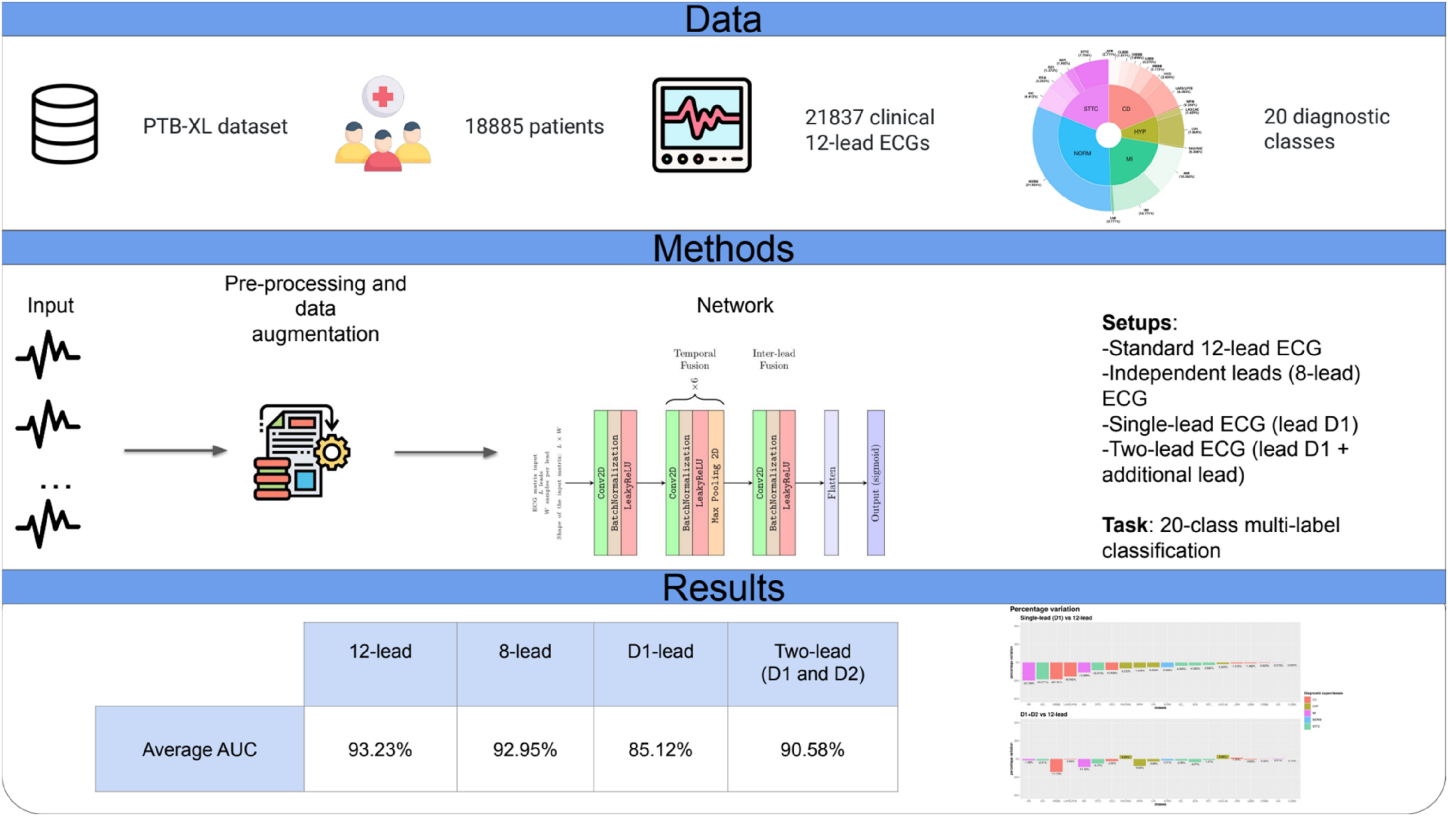
Graphical summary of the study.

The present study analyzed over 20,000 ECGs with 20 different abnormalities confirming the accuracy of a lightweight deep learning algorithm based on CNN (also with a very simplified architecture) and significantly increasing our understanding of the potential of AI-based ECG diagnostics. Figure 4 shows a graphical summary of the main findings of the study.

The main new finding was the demonstration that the D1 lead analyzed with the same CNN approach provided an overall accuracy that was only slightly inferior to that of the full 12-lead setup. For several ECG abnormalities, including important conditions such as AV block, complete left or right bundle branch block and LMI, the diagnostic yield of this single-lead approach was almost identical to that provided by the 12-lead standard setup.

Adding a second lead to D1 further reduced the performance gap with the 12-lead ECG setup. On average, the best AUC for a two-lead setup was achieved by combination D1 + D2: the simple combination of these two contiguous leads, by adding a second spatial axis exploring cardiac electrical activity, allowed to reduce the diagnostic gap by 64% (from a relative percentage difference of −7.8% to −2.8%). Notably, this approach also was capable of providing a marked increase in the accuracy of detecting myocardial ischemia and infarction of the inferior wall, a condition difficult to detect from the sole D1 lead. In addition, for some cases, such as the diagnoses of right and left atrial enlargement, the two-lead analysis outperformed the 12-lead setup, probably because it included the most informative lead (D2) for these alterations avoiding less informative leads potentially introducing noise. On the other hand, in terms of detection of myocardial ischemia and infarction of the anterior wall, the D1 + V2 setup overcame the D1 + D2 setup, due to the need, in these specific clinical scenarios, for a lead (such as V2), which spatially explores the site where the typical alterations for these conditions are found. Focusing on architecture, our proposed relatively lightweight neural network (36,000–96,000 trainable parameters) yielded similar results to those of more complex architectures [Attia et al. (6): 300,000 parameters; top-performing architecture by Strodthoff et al. (15): 3.5 million parameters]. In particular, we highlighted the fact that the present CNN model, in the 12-lead input setup, numerically outperformed the far more complex ResNet architecture proposed by Strodhtoff et al. for the same multi-label classification task (AUC: 0.932 vs. 0.931; please refer to Table 1). This high performance is due to the neural network design, tailored for this specific application, and to the extensive use of data augmentation, which also represents a significant step forward relative to previous studies.

The present findings have potentially large implications. As an example, even an acute myocardial infarction may potentially be detected by a single-lead recording. There is an enormous effort to reduce the time delay between the hospital door and the ECG execution in case of chest pain, applying refined triage strategies. However, the time spared, although statistically significant, is often very modest [1 min in the study by Su et al. (17)]. Thus, the delay from symptom onset to diagnostic ECG is the most actionable and likely the sole independent predictor of pre-discharge LVEF (18) and possibly of mortality (19). In addition, left bundle branch blocks (both complete and incomplete) and non-specific intraventricular conduction disturbances are accurately recognized by the AI-enabled single-lead ECG and, given their association with increased mortality (20), might prompt the need for further clinical investigations. The same is also true for atrioventricular blocks.

## Conclusions

AI-enabled ECG based on the sole D1 lead showed good performance in predicting cardiac abnormalities with a limited drop in AUC if compared to 12-lead ECG. Adding the additional lead D2 further reduces the performance gap. Altogether, our results prove that AI-enabled single or two-lead ECG analysis might be sufficient to detect cardiac abnormalities that have been classically diagnosed by 12-lead ECG. These results, paired with the relatively low complexity of our approach, lay the basis for extending the use of AI-enabled analysis of a reduced number of ECG recordings derived from the increasingly available wearable devices, potentially favoring a more accessible and large-scale screening of cardiac conditions. In a smartwatch, a D1-like lead is the default recording (obtained by keeping the watch on the left wrist and touching the crown with the right index), but a D2-like lead can be easily and reliably obtained by simply moving the watch to the left lower abdomen, still keeping the right finger on the crown (21). Precordial leads could also be obtained, but their recording appears more cumbersome. In this context, the recent work by Attia et al. (12) is noteworthy, as they effectively retrained a deep neural network originally designed for analyzing 12-lead ECGs to be compatible with single-lead ECGs derived from wearable devices (although for a specific task). However, our current experimental evidence is entirely based on leads from standard 12-lead ECGs, and not directly on single-lead recordings from wearable devices. We expect that reproducing our results on wearable devices would pose a set of unique challenges. For example, the increased impedance of a dry electrode on the wrist may lead to increased sampling noise while wearing the device at peripheral locations on the body might attenuate the signal amplitude, calling for robust noise filtering techniques. Together with the need to minimize the complexity due to the constrained processing capabilities, wearable devices represent a formidable challenge both in a medical and engineering sense, prompting further research in this direction.

## Data Availability

Publicly available datasets were analyzed in this study. This data can be found here: https://physionet.org/content/ptb-xl/1.0.1/.
