# Erratum: Convolutional neural network (CNN)-enabled electrocardiogram (ECG) analysis: a comparison between standard twelve-lead and single-lead setups

**DOI:** 10.3389/fcvm.2024.1396396

**Published:** 2024-03-14

**Authors:** Frontiers Production Office

**Affiliations:** Frontiers Media SA, Lausanne, Switzerland

**Keywords:** artificial intelligence, deep learning, electrocardiogram, single-lead, screening

An Erratum on: Convolutional neural network (CNN)-enabled electrocardiogram (ECG) analysis: a comparison between standard twelve-lead and single-lead setups By Saglietto A, Baccega D, Esposito R, Anselmino M, Dusi V, Fiandrotti A and De Ferrari GM (2024). Front. Cardiovasc. Med. 11:1327179. doi: 10.3389/fcvm.2024.1327179

Due to a production error, the first sentence of the Results paragraph in the abstract was incorrectly given as “The CNN based on single-lead ECG (D1) outperformed the one based on the standard 12-lead framework [with an average percentage difference of the area under the curve (AUC) of −8.7%].”.

This has been corrected to “The CNN based on single-lead ECG (D1) achieved satisfactory performance compared to the standard 12-lead framework (average percentage AUC difference: −8.7%).”

The publisher apologizes for this mistake. The original version of this article has been updated.

