# Supplementary material for: Convolutional neural network (CNN)-enabled electrocardiogram (ECG) analysis: a comparison between standard twelve-lead and single-lead setups
Source: Front Cardiovasc Med. 2024 Feb 15;11:1327179. doi: 10.3389/fcvm.2024.1327179 (PMC10901971; doi:10.3389/fcvm.2024.1327179)
Supplement: Supplementary file 2 [file Datasheet2.pdf]

Convolutional Neural Network (CNN)-enabled electrocardiogram (ECG) analysis: a comparison between standard twelve-lead and single-lead setups

Supplementary Material

Supplementary Methods

To improve the rigor of the study, we also tested a fine-tuned version of the trained network on two other external freely available datasets, the Georgia dataset[1] and the China dataset[2], confirming the findings on the PTB-XL dataset.

In particular, the ECGs in the Georgia dataset are of 10 seconds length with a sampling frequency of 500 Hz. We sub-sampled the ECGs to 100Hz and we rescaled the values to the same scale of the PTB-XL dataset. We have 10292 ECGs (80% train and 20% test), not considering classes with less than 10 ECGs, and 21 classes: 1st degree AV Block (IAVB), Atrial Fibrillation (AF), Atrial Flutter (AFL), Complete Right Bundle Branch Block (CRBBB), Incomplete Right Bundle Branch Block (IRBBB), Left Anterior Fascicular Block (LAnFB), Left Axis Deviation (LAD), Left Bundle Branch Block (LBBB), Low QRS Voltages (LQRSV), NonSpecific IntraVentricular Conduction disorder (NSIVCB), Premature Atrial Contraction (PAC), proLonged QT interval (LQT), Qwave Abnormal (QAb), Right Axis Deviation (RAD), Sinus Arrhythmia (SA), Sinus Bradycardia (SB), SiNus Rhythm (SNR), Sinus Tachycardia (STach), T wave abnormal (Tab), T wave Inversion (TInv), Ventricular Premature Beats (VPB).

The ECGs in the China dataset are from 6 to 60 seconds length with a sampling frequency of 500 Hz. We discarded all the ECGs with a length shorter than 10 seconds and we cropped 10 seconds ECGs for all those ECGs with a length greater than 10 seconds. We sub-sampled the ECGs to 100Hz and we rescaled the values to the same scale of the PTB-XL dataset. We have 2038 ECGs (80% train and 20% test), not considering classes with less than 10 ECGs, and 12 classes: 1st degree AV Block (IAVB), Atrial Fibrillation (AF), Atrial Flutter (AFL), Complete Right Bundle Branch Block (CRBBB), Incomplete Right Bundle Branch Block (IRBBB), Left Bundle Branch Block (LBBB), Premature Atrial Contraction (PAC), Sinus Arrhythmia (SA), Sinus Bradycardia (SB), SiNus Rhythm (SNR), Sinus Tachycardia (STach), T wave Abnormal (TAb).

We focused the attention only on three setups: single-lead (D1), two-lead (D1 + D2) and 12-lead.

For both the datasets, we substituted the last layer with a new classification layer and we fine-tuned the trained network in two steps (using the same data augmentation techniques presented in the main manuscript):

1. At first, we trained the new classifier for 10 epochs with a learning rate equal to 1e-4.
2. Secondly, we fine-tuned the whole network for 10 epochs with a learning rate equal to 1e-3.

Supplementary Table 1 and 2 show the average AUCs obtained on the Georgia test set and on the China test set that confirmed the findings on the PTB-XL dataset.

---

[1] https://www.kaggle.com/datasets/bjoernjostein/georgia-12lead-ecg-challenge-database

[2] https://www.kaggle.com/datasets/bjoernjostein/china-12lead-ecg-challenge-database

Moreover, we computed the challenge score of the PhysioNet Challenge 2021 (using the functions provided[3]) on the test set of the Georgia and China dataset comparing them to state-of-the-art values finding that they are consistent (please refer to Supplementary Table 3).

[3] https://github.com/physionetchallenges/evaluation-2021

Supplementary Tables

Supplementary Table 1. Average AUCs on Georgia test set (over 10 runs).

Classes	D1	D1 + D2	12 leads
IAVB	93.81	95.30	94.84
AF	86.24	89.42	89.35
AFL	81.47	83.09	85.21
CRBBB	95.67	96.34	97.61
IRBBB	81.36	87.15	95.22
LAnFB	74.64	96.82	96.33
LAD	71.17	96.61	96.31
LBBB	98.97	98.83	98.78
LQRSV	79.77	87.97	93.73
NSIVCB	75.58	80.22	87.62
PAC	63.2	64.83	66.46
LQT	77.96	80.02	85.25
QAb	71.60	79.49	86.47
RAD	91.02	92.53	96.17
SA	68.56	71.40	68.48
SB	96.37	96.81	96.96
SNR	87.12	89.66	91.66
STach	97.68	97.66	97.54
TAb	80.92	84.97	88.37
TInv	65.35	68.07	67.85
VPB	76.41	78.33	80.20
Average	81.66	86.45	88.59

Supplementary Table 2. **Average AUCs on China test set (over 10 runs).**

Classes	D1	D1 + D2	12 leads
IAVB	71.79	86.19	91.09
AF	85.74	94.99	95.82
AFL	70.07	60.87	74.94
CRBBB	91.98	95.93	98.32
IRBBB	71.15	80.24	90.4
LBBB	47.63	64.11	61.34
PAC	62.89	62.98	66.66
SA	90.11	79.08	76.86
SB	59.97	74.65	78.57
SNR	79.66	74.03	86.12
STach	91.75	94.21	95.6
TAb	33.99	43	60.46
Average	71.39	75.86	81.35

Supplementary Table 3. **Challenge scores on the Georgia and China test set (over 10 runs) compared to the literature.**

	D1	D1 + D2	12 leads
Our fine-tuned network on Georgia dataset	0.47	0.50	0.49
Our fine-tuned network on China dataset	0.27	0.35	0.36
[1]	-	0.44	0.49
[2]	-	0.58	0.58
[3]	-	0.39	0.35
[4]	-	0.48	0.49
[5]	-	0.52	0.52
[6]	-	0.49	0.52
[7]	-	0.45	0.48
[8]	-	0.47	0.49

Supplementary Table 4. Average specificity computed on the test set of the PTB-XL dataset (over 50 runs).

Classes	D1	D1 + D2	D1 + V1	D1 + V2	D1 + V3	D1 + V4	D1 + V5	D1 + V6	8 leads	12 leads	12 w/o aug
NORM	76.37	82.18	79.97	80.30	80.30	80.48	81.05	80.90	87.17	87.69	87.22
STTC	70.58	71.06	71.60	72.74	72.87	77.22	76.56	73.51	80.55	79.11	78.51
AMI	73.36	77.36	80.31	86.63	88.08	81.92	73.00	75.09	87.93	88.18	88.11
IMI	63.59	85.12	63.23	65.02	67.40	68.41	71.38	75.61	84.27	86.90	86.37
LAFB/LPFB	75.09	92.33	74.72	75.00	73.14	78.15	82.98	85.82	92.40	91.55	91.34
IRBBB	74.94	73.66	89.90	81.35	72.15	71.10	70.79	70.74	90.79	90.30	88.74
LVH	82.83	81.39	85.30	83.03	85.42	87.13	84.64	86.56	85.50	86.50	86.38
CLBBB	97.24	98.60	98.58	98.32	97.06	97.17	97.40	98.79	98.95	98.58	98.21
NST_	70.17	73.02	68.59	69.48	70.34	71.69	77.37	75.09	76.84	76.24	75.90
ISCA	77.75	79.54	78.38	75.75	77.27	77.84	79.49	79.45	80.15	81.55	79.52
CRBBB	96.20	97.31	98.55	97.52	97.12	96.65	96.53	97.04	98.28	98.2	98.03
IVCD	68.76	74.01	79.48	73.9	75.03	72.55	73.22	75.78	81.25	83.48	74.82
ISC_	87.24	85.45	85.86	83.77	83.49	85.79	88.39	89.08	89.23	88.77	88.52
_AVB	81.19	85.31	82.84	81.54	81.92	82.12	82.5	82.05	83.09	82.52	83.58
ISCI	56.93	79.74	62.43	66.53	64.7	68.14	79.64	74.53	82.46	85.05	83.7
WPW	93.32	91.06	91.26	91.21	92.6	90.68	90.96	88.52	88.91	89.55	86.87
LAO/LAE	70.46	71.81	70.54	66.7	65.7	69.38	72.73	70.93	71.19	71.33	73.16
ILBBB	94.51	94.7	93.68	93.5	91.37	93.42	93.41	91.18	89.27	90.19	85.2
RAO/RAE	81.96	90.22	79.09	79.16	85.75	86.29	86.03	82.37	86.07	89.48	85.39
LMI	97.54	99.23	97.41	98.4	98.53	98.74	98.78	99.18	96.23	98.08	91.88
Average	79.5	84.15	81.59	80.99	81.01	81.74	82.84	82.61	86.53	87.16	85.57

Supplementary Table 5. Average sensitivity computed on the test set of the PTB-XL dataset (over 50 runs).

Classes	D1	D1 + D2	D1 + V1	D1 + V2	D1 + V3	D1 + V4	D1 + V5	D1 + V6	8 leads	12 leads	12 w/o aug
NORM	90.37	93.79	90.31	90.28	91.77	92.81	92.22	91.98	93.55	93.74	93.25
STTC	78.34	83.75	78.42	82.25	83.80	83.10	85.35	85.43	84.4	86.32	85.52
AMI	80.96	81.03	86.36	90.88	88.34	83.92	84.71	84.98	92.91	93.06	91.82
IMI	70.97	88.61	76.85	73.87	73.79	74.79	76.74	76.21	88.71	90.95	91.15
LAFB/LPFB	70.44	94.94	78.63	73.59	75.94	79.01	81.18	82.21	95.26	95.30	93.99
IRBBB	62.91	70.76	92.21	86.19	68.14	69.07	69.50	75.32	91.61	91.83	90.16
LVH	82.46	84.02	84.55	84.53	83.45	83.92	87.58	88.73	89.78	88.73	91.19
CLBBB	95.89	96.04	96.52	96.59	97.04	96.74	97.04	96.19	96.11	95.70	96.00
NST_	87.22	88.47	88.04	88.75	86.12	84.75	83.41	84.67	87.69	88.35	87.29
ISCA	86.80	86.08	87.15	90.61	91.84	91.15	85.87	83.68	92.00	91.01	88.75
CRBBB	95.59	96.59	98.15	97.30	95.63	96.11	96.56	96.22	98.00	98.00	97.70
IVCD	60.12	64.51	58.15	64.42	59.01	59.43	58.06	57.91	61.82	59.85	69.70
ISC_	81.17	87.95	84.92	85.24	86.04	89.08	90.99	91.06	90.04	90.30	90.14
_AVB	91.37	93.12	90.76	92.37	91.98	92.02	93.00	93.22	93.17	92.34	90.83
ISCI	78.96	88.59	74.52	76.15	81.11	79.56	71.19	73.78	88.37	89.33	83.70
WPW	69.43	65.71	69.43	61.43	65.71	68.00	65.14	64.00	73.71	76.00	73.43
LAO/LAE	81.26	89.63	85.19	82.81	83.56	83.63	80.81	80.37	78.74	81.33	75.33
ILBBB	75.00	75.00	74.50	75.25	70.75	75.75	76.00	75.50	75.00	73.75	69.50
RAO/RAE	69.25	85.50	77.75	73.00	63.75	73.50	73.75	75.25	80.75	74.00	78.50
LMI	66.67	66.67	66.67	66.67	66.67	66.67	66.67	66.67	66.67	66.67	66.00
Average	78.76	84.04	81.95	81.61	80.22	81.15	80.79	81.17	85.91	85.83	85.20

Supplementary References

1 Natarajan, Annamalai, et al. "Convolution-free waveform transformers for multi-lead ECG classification." *2021 Computing in Cardiology (CinC)*. Vol. 48. IEEE, 2021.

2 *Nejedly, Petr, et al. "Classification of ECG using ensemble of residual CNNs with attention mechanism." 2021 Computing in Cardiology (CinC). Vol. 48. IEEE, 2021.*

3 *Pan, Lebing, et al. "MTFNet: a morphological and temporal features network for multiple leads ECG classification." 2021 Computing in Cardiology (CinC). Vol. 48. IEEE, 2021.*

4 Xiaoyu, L., et al. "Towards generalization of cardiac abnormality classification using ECG signal." Computing in Cardiology 2021 48 (2021): 2021.

5 Bruoth, Erik, et al. "A two-phase multilabel ECG classification using one-dimensional convolutional neural network and modified labels." 2021 Computing in Cardiology (CinC). Vol. 48. IEEE, 2021.

6 Vázquez, Cristina Gallego, et al. "Two will do: CNN with asymmetric loss, self-learning label correction, and hand-crafted features for imbalanced multi-label ECG data classification." 2021 Computing in Cardiology (CinC). Vol. 48. IEEE, 2021.

7 Suh, Jangwon, et al. "Learning ECG representations for multi-label classification of cardiac abnormalities." 2021 Computing in Cardiology (CinC). Vol. 48. IEEE, 2021.

8 Seki, Hiroshi, et al. "Reduced-lead ECG classifier model trained with DivideMix and model ensemble." 2021 Computing in Cardiology (CinC). Vol. 48. IEEE, 2021.
